# The bHLH1-DTX35/DFR module regulates pollen fertility by promoting flavonoid biosynthesis in *Capsicum annuum* L.

**DOI:** 10.1093/hr/uhac172

**Published:** 2022-08-03

**Authors:** Zhishuo Zhang, Yi Liu, Qiaoling Yuan, Cheng Xiong, Hao Xu, Bowen Hu, Huan Suo, Sha Yang, Xilin Hou, Fang Yuan, Zhenming Pei, Xiongze Dai, Xuexiao Zou, Feng Liu

**Affiliations:** Engineering Research Center of Education Ministry for Germplasm Innovation and Breeding New Varieties of Horticultural Crops, Key Laboratory for Vegetable Biology of Hunan Province, College of Horticulture, Hunan Agricultural University, Changsha 410125, China; College of Horticulture, Nanjing Agricultural University, Nanjing 210095, China; Engineering Research Center of Education Ministry for Germplasm Innovation and Breeding New Varieties of Horticultural Crops, Key Laboratory for Vegetable Biology of Hunan Province, College of Horticulture, Hunan Agricultural University, Changsha 410125, China; Longping Branch, Graduate School of Hunan University, Changsha 410125, China; Engineering Research Center of Education Ministry for Germplasm Innovation and Breeding New Varieties of Horticultural Crops, Key Laboratory for Vegetable Biology of Hunan Province, College of Horticulture, Hunan Agricultural University, Changsha 410125, China; Engineering Research Center of Education Ministry for Germplasm Innovation and Breeding New Varieties of Horticultural Crops, Key Laboratory for Vegetable Biology of Hunan Province, College of Horticulture, Hunan Agricultural University, Changsha 410125, China; Engineering Research Center of Education Ministry for Germplasm Innovation and Breeding New Varieties of Horticultural Crops, Key Laboratory for Vegetable Biology of Hunan Province, College of Horticulture, Hunan Agricultural University, Changsha 410125, China; Engineering Research Center of Education Ministry for Germplasm Innovation and Breeding New Varieties of Horticultural Crops, Key Laboratory for Vegetable Biology of Hunan Province, College of Horticulture, Hunan Agricultural University, Changsha 410125, China; Engineering Research Center of Education Ministry for Germplasm Innovation and Breeding New Varieties of Horticultural Crops, Key Laboratory for Vegetable Biology of Hunan Province, College of Horticulture, Hunan Agricultural University, Changsha 410125, China; Engineering Research Center of Education Ministry for Germplasm Innovation and Breeding New Varieties of Horticultural Crops, Key Laboratory for Vegetable Biology of Hunan Province, College of Horticulture, Hunan Agricultural University, Changsha 410125, China; College of Horticulture, Nanjing Agricultural University, Nanjing 210095, China; Engineering Research Center of Education Ministry for Germplasm Innovation and Breeding New Varieties of Horticultural Crops, Key Laboratory for Vegetable Biology of Hunan Province, College of Horticulture, Hunan Agricultural University, Changsha 410125, China; Engineering Research Center of Education Ministry for Germplasm Innovation and Breeding New Varieties of Horticultural Crops, Key Laboratory for Vegetable Biology of Hunan Province, College of Horticulture, Hunan Agricultural University, Changsha 410125, China; Engineering Research Center of Education Ministry for Germplasm Innovation and Breeding New Varieties of Horticultural Crops, Key Laboratory for Vegetable Biology of Hunan Province, College of Horticulture, Hunan Agricultural University, Changsha 410125, China; Engineering Research Center of Education Ministry for Germplasm Innovation and Breeding New Varieties of Horticultural Crops, Key Laboratory for Vegetable Biology of Hunan Province, College of Horticulture, Hunan Agricultural University, Changsha 410125, China; Engineering Research Center of Education Ministry for Germplasm Innovation and Breeding New Varieties of Horticultural Crops, Key Laboratory for Vegetable Biology of Hunan Province, College of Horticulture, Hunan Agricultural University, Changsha 410125, China

## Abstract

High pollen fertility can ensure the yield and efficiency of breeding work, but factors that affect the fertility of pepper pollen have not been studied extensively. In this work, we screened the reduced pollen fertility 1 (*rpf1*) mutant of *Capsicum annuum* with reduced pollen fertility and yellow anthers from an EMS (ethyl methanesulfonate)-mutagenized pepper population. Through construction of an *F*_2_ population followed by BSA (bulked segregant analysis) mapping and KASP genotyping, we identified *CabHLH1* as a candidate gene for control of this trait. A G → A mutation at a splice acceptor site in *CabHLH1* causes a frameshift mutation in the mutant, and the translated protein is terminated prematurely. Previous studies on CabHLH1 have focused on the regulation of flavonoid synthesis. Here, we found that *CabHLH1* also has an important effect on pollen fertility. Pollen vigor, anther flavonoid content, and seed number were lower in *CabHLH1*-silenced pepper plants, whereas anther H_2_O_2_ and MDA (malondialdehyde) contents were higher. RNA-seq analyses showed that expression of the flavonoid synthesis genes *DFR*, *ANS*, and *RT* was significantly reduced in anthers of *CabHLH1*-silenced plants and *rpf1* plants, as was the expression of *DTX35*, a gene related to pollen fertility and flavonoid transport. Yeast one-hybrid and dual-luciferase reporter assays showed that CabHLH1 can directly bind to the promoters of *DTX35* and *DFR* and activate their expression. These results indicate that CabHLH1 regulates reactive oxygen species homeostasis by promoting the synthesis of anther flavonoids and acts as a positive regulator of pepper pollen fertility.

## Introduction

As the male gametophyte, pollen carries and transmits genetic information from the male parent, and it plays a key role in the processes of sexual reproduction and seed formation in higher plants. High pollen fertility can ensure fruit yield and the efficiency of breeding work. Factors that affect pollen fertility include temperature [1], moisture [2], and chemical substances [3].

Flavonoids are an important class of secondary metabolites that exist in different tissues and organs of plants. They consist mainly of anthocyanins, flavan-3-ols, flavanones, flavonols, flavones, and isoflavonoids [4]. Flavonoids play a vital role in plant reproduction. Flavonols can maintain steady-state levels of reactive oxygen species (ROS) in plants under high-temperature stress, thereby ensuring that plants can complete reproductive activities normally [5]. When corn and petunia lack flavonols, their pollen does not germinate normally, but when kaempferol is added to the germination medium or stigma during pollination, the pollen regains its function [6]. When flavonoid content is reduced in *chalcone synthase* (*CHS*) RNAi tomato plants, pollen development and pollen tube elongation appear abnormal; plants show partial or complete male sterility, fruits are small and developmentally delayed, seed development is severely impaired, and parthenocarpy appears [7]. In *Arabidopsis*, the transporter encoded by the flavonoid *DTX35* gene in flowers is indispensable for the transport of flavonols and is expressed in cells of the stigma, anthers, and nectaries. *DTX35* mutant plants showed a reduction in glycosylated flavonol content; their anthers did not dehisce normally, and their pollen fertility was diminished [8]. The *Arabidopsis MYB21* mutant accumulates excess ROS in the stamens, but treatment with exogenous kaempferol or overexpression of the structural gene *FLS1* rescues the fertility of the mutant stamens [9].

The biosynthetic pathways of plant flavonoids and their key genes have been reported in detail [10]. Phenylalanine is converted to coumaric acid-CoA through the activities of phenylalanine ammonia lyase (PAL), cinnamic acid-4-hydroxylase (C4H), and 4-coumaric acid-CoA ligase (4CL). Successive enzymatic steps catalyzed by CHS, chalcone isomerase (CHI), flavanone-3-hydroxylase (F3H), flavonoid 3′-hydroxylase (F3′H), and flavonoid 3′5′-hydroxylase (F3′5′H) result in the formation of dihydroflavonols from coumaric acid-CoA and malonyl-CoA, and dihydroflavonols are then converted to flavonols by flavonol synthase (FLS). Flavonols may be further modified by glycosyltransferase/rhamnosyltransferase (GT/RT) to increase their stability [10]. In addition, dihydroflavonol 4-reductase (DFR) and anthocyanin synthase (ANS) catalyze the synthesis of various types of anthocyanins, which are finally modified into more stable anthocyanin glycosides by GT/RT [10]. However, there are few studies on the key transcription factors that regulate the structural genes of flavonoid synthesis.

Many *basic helix–loop–helix* (*bHLH*) transcription factors have been reported to affect anther fertility. In petunia, anther fertility is significantly reduced when *PhMYC2* regulates the overexpression of the anther-specific *GRP* gene [11]. A mutant in the *bHLH* transcription factor gene *AMS* exhibits delayed degradation of tapetal cells and impaired pollen wall formation, and a mutant in the ABC transporter gene *WBC27* shows a similar phenotype. Quantitative chromatin immunoprecipitation (qChIP)–PCR and electrophoretic mobility shift assay (EMSA) experiments showed that AMS directly regulates the expression of *WBC27*. These results show that the *AMS* gene and its regulatory network have a very important role in normal pollen function [12]. The tapetum of a mutant in the bHLH transcription factor gene *UDT1* is unable to differentiate and continues to vacuolate; the mutant anthers are white and lack fertile pollen grains, and the inflorescence cannot produce normal seeds [13]. EAT1 directly regulates the expression of *OsAP25* and *OsAP37*, two aspartic proteases that promote plant cell death. The expression of *OsAP25* and *OsAP37* was significantly reduced in an *EAT1* mutant, and the programmed death of tapetum cells was delayed, resulting in abnormal pollen development and a sterile phenotype [14]. However, these studies did not examine whether *bHLH* transcription factors affect pollen fertility by regulating flavonoid synthesis. Therefore, we performed further research on this issue.

Pepper (*Capsicum annuum* L.) is an economically important crop that is widely cultivated throughout the world and is one of the main vegetable crops in China. Most pepper varieties have high pollen fertility and purple anthers. Here, we obtained the pepper mutant *rpf1* with significantly reduced pollen fertility and yellow anthers. We identified *CabHLH1* as a candidate gene for control of this trait through bulked segregant analysis (BSA) localization and KASP typing analysis. Silencing of *CabHLH1* reduced the fertility of pepper pollen and the content of anther flavonoids, promoted the accumulation of active oxygen species, and reduced seed number. Pollen fertility was significantly restored after spraying with the ROS scavenger diphenyleneiodonium (DPI). Yeast one-hybrid (Y1H) and dual-luciferase (LUC) reporter assays verified that CabHLH1 affects pollen fertility by regulating the expression of flavonoid synthesis and transport genes. This study shows that CabHLH1-mediated flavonoid synthesis in pepper anthers is essential for maintaining ROS homeostasis and ensuring pollen fertility.

## Results

### Isolation and phenotypic characteristics of the *rpf1* mutant

The chili pepper backbone parent ‘8214’ was used as the material for EMS mutagenesis. The specific steps followed previous research methods with slight modifications [32]. Five thousand seeds of ‘8214’ treated with EMS (0.6%) and 200 control seeds were sown. After culture for 30 days in the greenhouse, plants were transplanted into plastic greenhouses. Seeds were saved after selfing of an individual plant, and seeds harvested from each individual plant constituted one line, i.e. the *M*_1_ line. Twenty peppers were planted per line. After flowering, mutants with yellow anthers, low flavonoid content, and low pollen vigor (nearly half of the pollen grains were stained yellow with I_2_-KI) were selected and harvested to obtain the *M*_2_ line. The *M*_3_ line was obtained by the same method as that used for the *M*_2_ line, the *M*_3_ line was planted, and mutant plants with stable inheritance were harvested to obtain the *rpf1* mutant.

**Figure 1:**
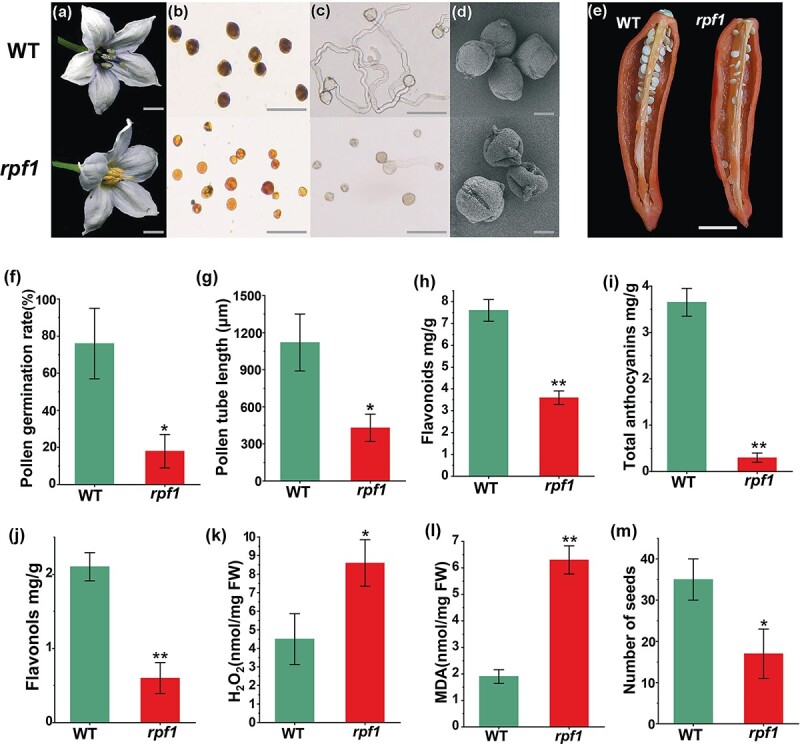
Phenotypes of WT and reduced pollen fertility (*rpf1*) mutant plants. **a** Flower phenotypes of WT and *rpf1*. Scale bars, 1 cm. **b** Pollen viability of WT and *rpf1*. Pollen grains in WT were stained black by KI-I_2_; *rpf1* pollen grains were stained golden yellow. Scale bars, 100 μm. **c** Pollen grains of WT and *rpf1* were germinated *in vitro*. Scale bars, 100 μm. **d** SEM images of WT and *rpf1* pollen grains. Scale bars, 10 μm. **e** Seeds of WT and *rpf1*. Scale bar, 1 cm. **f** Pollen germination rate *in vitro*. **g** Pollen-tube length. **h** Flavonoid content. **i** Total anthocyanin content. **j** Flavonol content. **k** H_2_O_2_ content. **l** MDA content. **m** Quantification of seed set. Error bars indicate mean ± standard error (*n* = 6) in **f** and **g**, mean ± standard error (*n* = 3) in **h**–**l**, and mean ± standard error (*n* = 20) in **m**. ^*^P < .05, ^**^P < .01.

WT ‘8214’ has purple anthers, whereas the *rpf1* mutant has yellow anthers (Fig. 1a). When pollen was stained with I_2_-KI solution, the WT pollen grains were fuller and darker, whereas the mutant pollen grains had lighter staining, inconsistent size, and lower pollen vigor (Fig. 1b). *In vitro* pollen germination experiments were performed, and pollen germination rate and pollen tube length were significantly lower in the *rpf1* mutant than in the WT (Fig. 1c, f, and g). We further observed pollen morphology by scanning electron microscopy. The WT pollen was plump and spherical, and its outer wall was smooth. The outer wall of the mutant pollen was inverted, and there was a clear lack of swelling (Fig. 1d). The contents of anthocyanins, flavonoids, and flavonols were reduced in anthers of the *rpf1* mutants (Fig. 1h–j), the contents of H_2_O_2_ and MDA were increased (Fig. 1k and i), and the number of fruit seeds was reduced (Fig. 1e and m). Flavonoids often affect the anther cuticle. The anthers of open flowers were observed by scanning electron microscopy. The epidermal cells of WT anthers were plump, neatly arranged, and compact. However, the epidermal cells of *rpf1* anthers were shriveled, and most of the cells were irregularly shrunken (Supplementary Data Fig. S1).

To further explore cytological defects during anther development, cross-sections of WT and *rpf1* anthers at different stages were observed, and no significant differences in anther structure were found (Supplementary Data Fig. S2). Pollen development was normal at the pollen mother cell, tetrad, and mononucleate microspore stages. The pollen mother cells underwent meiosis in the anther chamber to produce tetrads. After the disintegration of the tapetum, the microspores were released from the tetrad and dispersed in the anther sacs. In WT mature anthers, most mononucleate microspores developed into mature pollen. However, many immature pollen grains were present in *rpf1* anthers. Transmission electron microscopy showed that immature pollen was in the mononucleate microspore stage, suggesting that the development of mononucleate microspores into mature pollen was disturbed (Supplementary Data Fig. S3).

### Comparative transcriptomic analysis of ‘8214’ and *rpf1*

**Figure 2:**
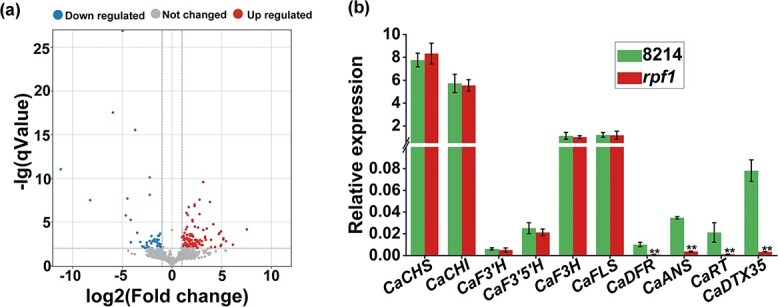
Analysis of DEGs in WT and *rpf1* libraries. **a** Volcano plot of DEGs. Red points indicate upregulated genes, green points indicate downregulated genes. **b** Identification of DEGs related to flavonoid synthesis by qRT–PCR. Values are mean ± standard error (*n* = 3). ^**^P < .01.

To investigate the reasons for differences in pollen fertility and anther flavonoid content between the WT and the *rpf1* mutant, we performed RNA-seq transcriptomic analysis on WT and *rpf1* anthers. Compared with the WT, a total of 161 DEGs were found in *rpf1*: 46 downregulated and 115 upregulated (Fig. 2a; Supplementary Data Table S2). GO annotation and enrichment analysis assigned biological process, cellular component, and molecular function GO terms to the DEGs (Supplementary Data Fig. S4a; Supplementary Data Table S3). In the molecular function category, the DEGs were enriched mainly in transferase activity and transmembrane transporter activity. In the biological process category, the DEGs were enriched mainly in flavonoid biosynthesis process, secondary metabolism process, vegetative meristem growth, flower morphogenesis, and other biological processes. In the cellular component category, the DEGs were enriched mainly in vacuole, vacuole membrane, apoplast, and extracellular region. We also performed KEGG enrichment analysis on the DEGs to further study the metabolic pathways in which they participate (Supplementary Data Fig. S4b; Supplementary Data Table S4). The DEGs were enriched mainly in metabolic pathways, MAPK signaling pathways, flavonoid biosynthesis, phenylalanine metabolism, biosynthesis of secondary metabolites, glutathione metabolism, and other pathways.

We analyzed the transcriptome data of structural genes that are involved in flavonoid biosynthesis. The expression levels of upstream genes in the pathway (*CHS*, *CHI*, *F3H*, *F3′5′H*, and *F3′H*) did not show significant differences in expression between the genotypes. By contrast, downstream genes (*DFR*, *ANS*, and *RT*) showed significantly higher expression levels in WT anthers than in mutant anthers (Supplementary Data Table S2). More importantly, we found that expression of *DTX35*, which has previously been reported to influence pollen fertility and flavonol transport, was significantly reduced in the mutant [33].

The expression levels of genes involved in cell wall macromolecule catabolic process and plant hormone signal transduction also differed significantly between WT and mutant anthers (Supplementary Data Tables S3 and S4). For example, *BXL4* (*Capana03g001860*) and *BXL1* (*Capana03g001861*) have both xylosidase and arabinosidase activity and participate in cell wall remodeling [34, 35]. *Endo-β-mannanase* (*Capana01g002420*) promotes the breakdown of mannan in the cell wall by hydrolyzing it. Their expression levels were significantly reduced in *rpf1* anthers. This may be the reason for the morphological changes in the epidermal cells of mutant anthers. In addition, recent studies have shown that the auxin signalling pathway regulates pollen mitotic division [36]. The expression levels of genes involved in plant hormone signal transduction were higher in mutant than WT anthers just prior to dehiscence. These included three genes (*Capana04g000808*, *Capana08g001238*, and *Capana02g003021*) encoding auxin-responsive protein 9, auxin-responsive protein 16, and indole-3-acetic acid-amido synthetase GH3.1, respectively. They promote the accumulation of auxin, suggesting that mutant anther development is relatively delayed at this time. Therefore, the mutants release immature pollen after flowering. We selected these genes for qRT–PCR verification of the transcriptome data, and their expression patterns were consistent with the RNA-seq results (Supplementary Data Table S2; Supplementary Data Fig. S5).

**Table 1:** Genetic analysis of the *rpf1* mutant.

Generations	Total	WT	Mutant	Segregation ratio	*χ* ^2^/*P* value
*P*_1_(‘8214’)	60	60	0		
*P*_2_(*rpf1*)	60	0	60		
*F*_1_	105	105	0		
BC_1_(*F*_1_ × *rpf1*)	103	54	49	1.06:1	0.24/0.62
*F*_2_	571	432	139	3.11:1	0.13/0.72

### Mapping of *Carpf1*, a candidate gene for the regulation of pollen fertility in pepper

All members of the *F*_1_ population obtained from a cross between ‘8214’ and *rpf1* showed the WT phenotype. A *χ*^2^ test of phenotypes in the BC_1_ population obtained by backcrossing *F*_1_ to *rpf1* showed that the ratio of WT to mutant phenotypes was close to 1:1 (*χ*^2^ = 0.24 < *χ*^2^_0.05, 1_ = 3.84). *F*_1_ selfing was used to construct an *F*_2_ segregation population. In the population of 571 *F*_2_ individuals, 139 progeny had mutant phenotypes, in line with the expected Mendelian segregation ratio of 3:1 for a single-gene trait (*χ*^2^ = 0.13 < *χ*^2^_0.05, 1_ = 3.84), indicating that the phenotype of *rpf1* was controlled by a single recessive nuclear gene (Table 1; Supplementary Data Table S5).

We selected 30 WT individuals and 30 mutant individuals from the *F*_2_ population, constructed two DNA pools, and performed mixed pool sequencing, resulting in a total of 120 Gb of data. The sequencing depths of the WT and mutant-type pools were 21.60× and 20.47×, respectively, covering >98% of the genome. After filtering, 2246 differential SNPs were obtained, and a peak harboring 7 SNPs located an 8.31-Mb candidate region on Chr01 (859 016–9 170 311) was selected for further analysis.

**Figure 3:**
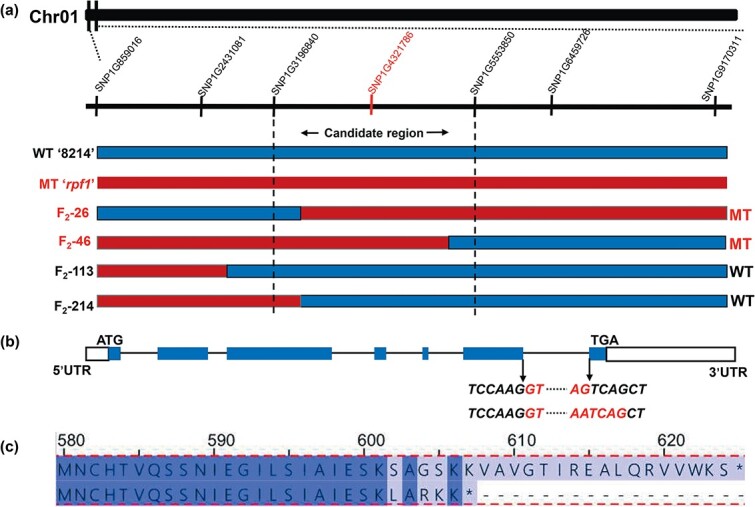
Identification of the gene conferring the reduced pollen fertility phenotype. **a** Linkage analysis of the *F*_2_ population using KASP markers confirmed the causative SNP. The SNP marked with red is the only SNP co-segregating with the reduced pollen fertility phenotype. *F*_2_-26 and *F*_2_-42 had mutant phenotypes (marked with red). *F*_2_-113 and *F*_2_-214 had wild phenotypes (marked with black). **b** Structure of predicted *CabHLH1* and the coding sequence alignment of CabHLH1 in ‘8214’ and *rpf1*. White boxes, blue box, and solid line represent 5′ and 3′UTR, exons, and introns, respectively. **c** Protein sequence alignment of CabHLH1 in ‘8214’ and *rpf1*.

**Figure 4:**
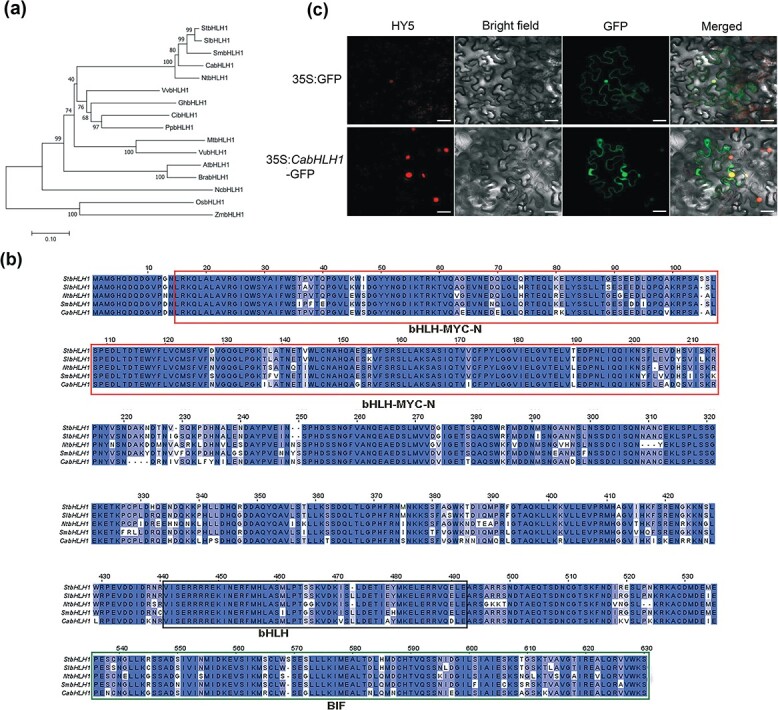
Characterization and subcellular localization of CabHLH1. **a** Phylogenetic analysis of 16 plants’ amino acid sequences of bHLH1. **b** Sequence alignment of bHLH1 sequences. The bHLH-MYC_N, HLH, and BIF domains are indicated by the lines around the sequences. **c** Subcellular localization of the CabHLH1 protein in *N. benthamiana* leaves. 35S:GFP was used as a control. HY5 was used as a nuclear marker. Scale bar, 15 μm.

To further narrow down the candidate SNPs, we used the *F*_2_ population to perform KASP genotyping analysis of the SNPs, and we found that SNP4321786 co-segregated with the *F*_2_ WT and mutant phenotypes (Fig. 3a). However, other SNP sites in the *F*_2_ population had recombinant individuals whose genotype and phenotype did not match (Supplementary Data Table S6). Therefore, we narrowed the candidate interval on Chr01 down to ~2.36 Mb, between the two markers SNP3196840 and 5553850. In this interval, only SNP4321786 is a non-synonymous mutation, and its G → A mutation at a splice acceptor site causes a frameshift mutation that results in the premature termination of *Capana01g000256* translation (Fig. 3b and c).

### Homology, subcellular localization, and expression pattern of CabHLH1

The Capana01g000256 protein sequence was compared with homologous protein sequences from other species such as tomato and *Arabidopsis* and was found to be closely related to proteins such as SlbHLH1 and StbHLH1 (Fig. 4a). We therefore named the pepper protein CabHLH1. We annotated the protein sequence of Capana01g000256 in the NCBI database and found that it encodes a bHLH transcription factor that regulates the synthesis of flavonoids and contains a bHLH-MYC_N domain, an HLH domain, and a BIF domain (Fig. 4b). Previous studies have shown that deletion of the BIF domain significantly reduces bHLH transcriptional activation activity and causes male sterility in watermelon [37]. These results indicate that the gene *Capana01g000256* containing SNP4321786 is a candidate gene for the mutant phenotype. To determine the subcellular location of CabHLH1, we used *Agrobacterium*-mediated transformation to transiently express 35S:CabHLH1-GFP in tobacco epidermal cells, with 35S:GFP transformed cells as controls. Both the plasma membrane and nucleus of tobacco cells showed green fluorescence (Fig. 4c), indicating that CabHLH1 is located in these two cellular regions.

**Figure 5:**
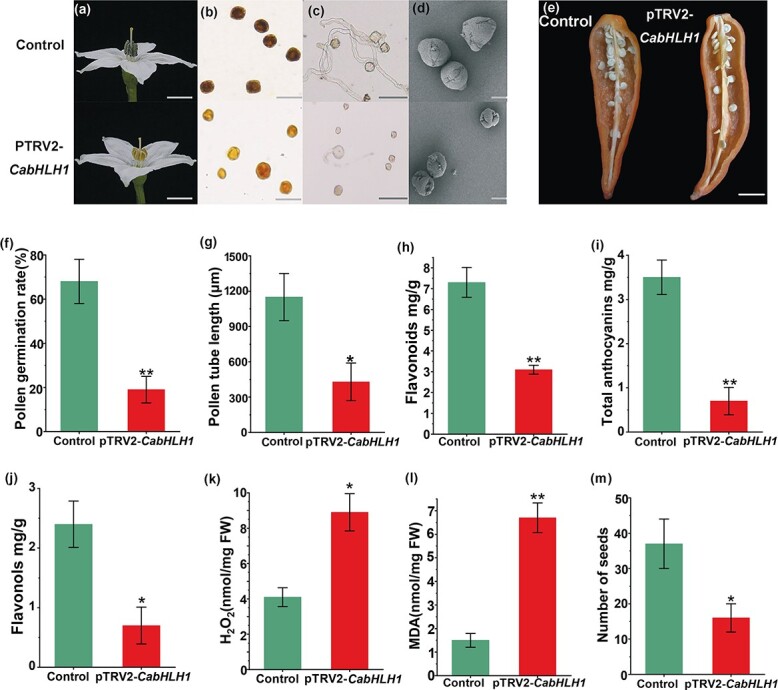
Phenotypes of control and pTRV2*-CabHLH1* plants. **a** Flower phenotypes of control and pTRV2-*CabHLH1* plants. Scale bars, 1 cm. **b** Pollen viability of control and pTRV2-*CabHLH1* plants. Pollen grains in control plants were stained black by KI-I_2_. pTRV2-*CabHLH1* plants were stained golden yellow. Scale bar, 100 μm. **c** Pollen grains of control and pTRV2-*CabHLH1* plants were germinated *in vitro*. Scale bar, 100 μm. **d** SEM images of control and pTRV2-*CabHLH1* pollen grains. Scale bar, 10 μm. **e** Seeds in control and pTRV2-*CabHLH1* fruits. Scale bar, 1 cm. **f** Pollen germination rate. **g** Pollen-tube length. **h** Flavonoid content. **i** Total anthocyanin content. **j** Flavonol content. **k** H_2_O_2_ content. **l** MDA content. **m** Quantification of seed set. Error bars indicate mean ± standard error (*n* = 6) in **f** and **g**, mean ± standard error (*n* = 3) in **h**–**l**, and mean ± standard error (*n* = 20) in **m**. ^*^P < .05, ^**^P < .01.

qRT–PCR analysis showed that the expression of *CabHLH1* was significantly higher in buds than in leaves, roots, stems, pericarp, seeds, and placentas (Supplementary Data Fig. S6a). The expression of *CabHLH1* was also determined in anthers at the pollen mother cell, tetrad, mononucleate microspore, and mature pollen stages. *CabHLH1* expression was highest in the mononucleate microspore stage (Supplementary Data Fig. S6b). To further validate the expression of *CabHLH1* in pepper anthers, we performed *in situ* hybridization to examine its expression in WT anthers at four developmental stages. The maximum hybridization signal was seen in the mononucleate microspore stage (Supplementary Data Fig. S6c–f). By contrast, weak hybridization signals were observed during the microspore mother cell stage. This result suggests that *CabHLH1* plays a role in the development of mononucleate microspores into mature pollen in anthers, consistent with the pollen development defect of *rpf1*. These results further confirmed that *CabHLH1* was the target gene.

### *CabHLH1* silencing reduces pepper anther flavonoid content and pollen fertility

To study how CabHLH1 affects pollen fertility, virus-induced gene silencing (VIGS) technology was used to silence *CabHLH1* in peppers. Four weeks after injection of peppers with *Agrobacterium* carrying pTRV2:*CabHLH1*, the expression of *CabHLH1* in anthers was significantly reduced, confirming that *CabHLH1* was successfully silenced (Supplementary Data Fig. S7). Compared with the controls, pTRV2:*CabHLH1* plants showed significant changes in anther colour, pollen vigor, pollen germination rate, and pollen tube length (Fig. 5a–c, f and g) and their pollen walls were sunken (Fig. 5d), the contents of anthocyanins, flavonoids, and flavonols in their anthers were reduced, and the contents of H_2_O_2_ and MDA were increased (Fig. 5h–l). The number of seeds was also reduced (Fig. 5e and m). We next analyzed the expression of genes in the anther flavonoid pathway by qRT–PCR in *CabHLH1*-silenced and control plants. The expression levels of upstream genes in the pathway (*CHS*, *CHI*, *F3H*, *F3′5′H*, and *F3′H*) did not differ significantly between the genotypes. By contrast, the expression levels of downstream genes (*DFR*, *ANS*, *RT*, and *DTX35*) were significantly lower in pTRV2:*CabHLH1* anthers than in control anthers (Supplementary Data Fig. S7), consistent with differences in the parental transcriptome data. During the pre-bloom period, DPI (an ROS inhibitor) was sprayed daily onto flower buds of the *CabHLH1*-silenced plants, and pollen fertility was assessed after flowering. Pollen vigor was significantly enhanced by DPI spraying, and the pollen germination rate and number of fruit seeds increased (Supplementary Data Fig. S8).

### CabHLH1 binds to the promoters of *DTX35* and *DFR* to promote their transcription

**Figure 6:**
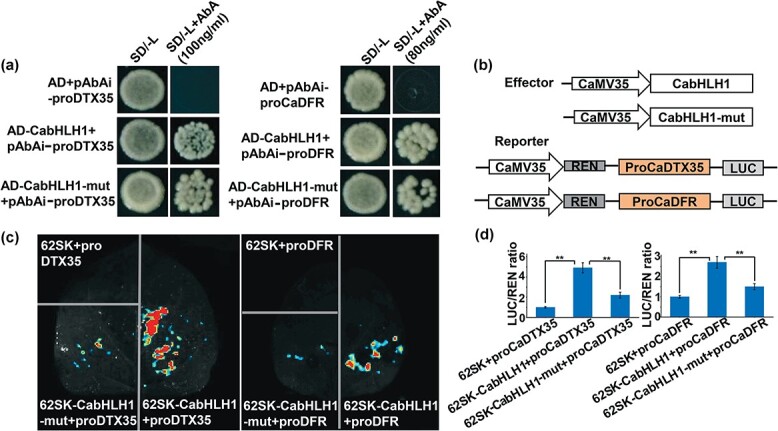
CabHLH1 binds to the promoters of *CaDTX35* and *CaDFR* to promote their transcription*.***a** DNA-binding potential of CabHLH1 with the promoters of *CaDTX35* and *CaDFR* in yeast. SD/−L, medium lacking leucine. **b** Schematic of the vector used for the dual-LUC experiment. **c** LUC images of tobacco leaves after transient infiltration. **d** Ratio of LUC to REN activity. Data are the mean ± standard deviation of six replicates. ^**^P < .01.

Based on the RNA-seq and qRT–PCR results, CabHLH1 appeared to affect the expression of multiple downstream genes in the flavonoid synthesis pathway. To further investigate the relationship between CabHLH1 and flavonoid biosynthesis genes, we performed Y1H assays to identify possible target genes. Previous studies have shown that bHLH transcription factors regulate target genes by binding to the G-box element cassette in their promoters. The promoters of *DTX35* and *DFR* both include G-box elements. The yeast strain Y1H Gold was co-transformed with pAbAi-*DTX35*, pAbAi-*DFR*, or a negative control and AD-CabHLH1 or AD-CabHLH1-mut. Except for the negative control, all transformants survived on selective medium lacking leucine and containing AbA. This result showed that CabHLH1 binds to the promoters of *DTX35* and *DFR* in yeast cells, and the mutation of the BIF domain did not affect its binding (Fig. 6a). The dual-LUC system was used to determine whether CabHLH1 could activate the expression of structural genes. As shown in the figure, the ratio of LUC to REN in tobacco leaves injected with 62SK-CabHLH1 and pro*DTX35*-LUC increased 3.3-fold relative to that in the negative control (62SK and pro*DTX35*-LUC). Compared with the negative control (62SK and proDFR-LUC), the LUC to REN ratio increased 1.6-fold in tobacco leaves injected with 62SK-CabHLH1 and pro*DFR*-LUC. However, when 62SK-CabHLH1 was replaced by 62SK-CabHLH1-mut, LUC activity was significantly reduced, and the transcriptional activation of the structural genes was significantly lower (Fig. 6b–d). All these data indicate that CabHLH1 directly binds to the promoters of *DTX35* and *DFR* and activates their expression in pepper anthers.

**Figure 7:**
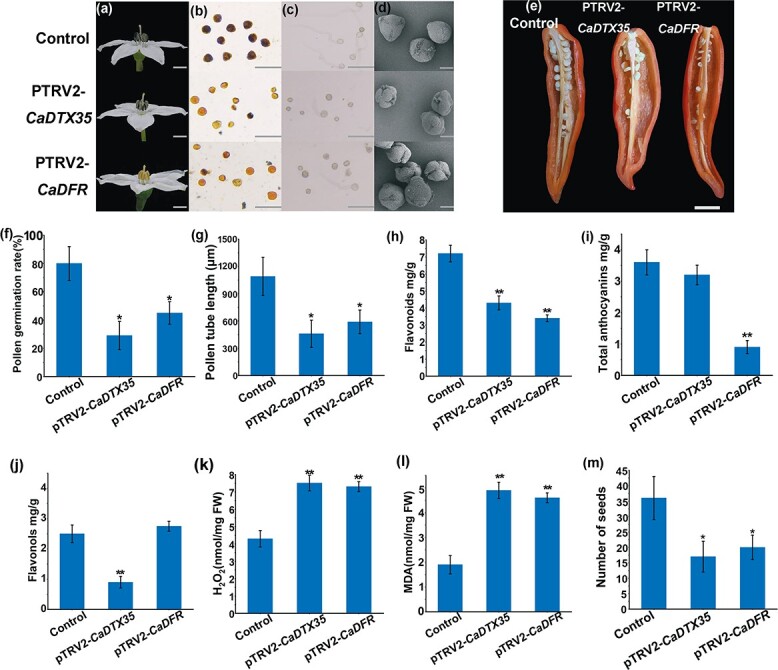
Phenotypes of control, pTRV2*-CaDTX35*, and pTRV2*-CaDFR* plants. **a** Flower phenotypes of control, pTRV2-*CaDTX35*, and pTRV2-*CaDFR* plants. Scale bars, 1 cm. **b** Pollen viability of control, pTRV2-*CaDTX35*, and pTRV2-*CaDFR* plants. Scale bars, 100 μm. **c** Pollen grains of control, pTRV2-*CaDTX35*, and pTRV2-*CaDFR* plants were germinated *in vitro*. Scale bars, 100 μm. **d** SEM images of pollen grains. Scale bars, 10 μm. **e** Seeds in fruits. Scale bar, 1 cm. **f** Pollen germination rate *in vitro*. **g** Pollen-tube length. **h** Flavonoid content. **i** Total anthocyanin content. **j** Flavonol content. **k** H_2_O_2_ content. **l** MDA content. **m** Quantification of seed set. Error bars indicate mean ± standard error (*n* = 6) in **f** and **g**, mean ± standard error (*n* = 3) in **h**–**l**, and mean ± standard error (*n* = 20) in **m**. ^*^P < .05, ^**^P < .01.

### *DTX35* or *DFR* silencing reduces pepper pollen fertility and anther flavonoid content

**Figure 8:**
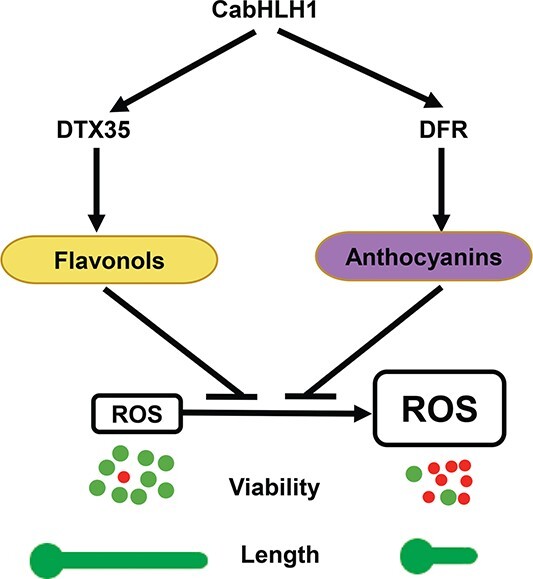
CabHLH1 regulates ROS homeostasis by promoting the synthesis of anther flavonoids and acts as a positive regulator of pepper pollen fertility. Green dots represent viable pollen grains, while red dots are dead grains

We also silenced *DTX35* and *DFR* in pepper by the VIGS method. The expression levels of *DTX35* and *DFR* were decreased in the anthers of the two silenced plants, respectively (Supplementary Data Fig. S9). The anther anthocyanin content of *DTX35*-silenced plants did not differ significantly from that of the controls. The anthers were still purple, but the flavonol content was significantly lower, the pollen vigor and germination rate were significantly reduced, the H_2_O_2_ and MDA contents were increased, and the number of seeds was reduced (Fig. 7a–m). Compared with the controls, anther anthocyanin content was significantly reduced in *DFR*-silenced plants, the flavonol content did not change significantly, the contents of H_2_O_2_ and MDA increased, and pollen vigor, germination rate, and number of seeds decreased (Fig. 7a–m). These results are consistent with a role for *DTX35* in the transport of flavonol compounds. Overall, our findings show that CabHLH1 maintains ROS homeostasis by regulating the synthesis and transport of flavonoids, thereby ensuring normal pollen activity (Fig. 8).

## Discussion

Pollen fertility is a very important plant trait that can directly affect seed setting rate and yield. Flavonoids play a vital role in maintaining ROS homeostasis and regulating normal pollen development. Their phenolic ring portion is sufficient to accept electrons and thus perform an antioxidant function [38]. In this study, we characterized the pepper EMS mutagenesis-derived *rpf1* mutant with significantly reduced pollen fertility and found that its anther flavonoid content was significantly lower than that of the WT. Through BSA sequencing and KASP typing detection, we located the target gene at the beginning of chromosome 1; it belonged to the bHLH family of transcription factors and was named *CabHLH1*. The mutant and *CabHLH1*-silenced plants both showed reduced pollen fertility, decreased expression of genes associated with flavonoid synthesis and transport, decreased flavonoid content, increased active oxygen accumulation, and lower seed numbers. Y1H and dual-LUC reporter assays confirmed that CabHLH1 affects pollen fertility by regulating the expression of flavonoid synthesis and transport genes.

In plants, bHLH transcription factors have been reported to regulate pollen fertility and flavonoid synthesis [11, 39, 40]. However, there has been little research on whether these two processes are correlated. Compared with the control plants, *CabHLH1*-silenced plants showed significantly reduced pollen fertility, significantly lower contents of anther anthocyanins and flavonols, higher accumulation of H_2_O_2_ and MDA, and fewer seeds. After DPI was sprayed onto flower buds, the pollen fertility of *CabHLH1*-silenced plants was restored. Previous studies have shown that excessive accumulation of ROS in anthers can cause pollen abortion [5], and flavonoids are important for the maintenance of ROS homeostasis [38]. CabHLH1 maintains ROS homeostasis by regulating the synthesis and transport of flavonoids, thereby ensuring normal pollen activity. qRT–PCR analysis showed that the expressions of downstream structural genes in the flavonoid biosynthetic pathway (*DFR*, *ANS*, and *RT*) and the flavonol transport gene *DTX35* were significantly reduced in anthers of *CabHLH1*-silenced plants. Transcription factors from the bHLH and MYB families regulate the expression of genes encoding key enzyme such as *FLS*, *CHS*, and *CHI*, which play important roles in regulating the biosynthesis and tissue accumulation of flavonols [41, 42]. However, there have been very few studies on the transcription factors that regulate the expression of *DTX35*. Through Y1H and dual-LUC reporter assays, we verified that CabHLH1 can directly bind to the *CaDTX35* promoter and regulate its expression, thereby affecting the synthesis of flavonols and revealing a new mechanism for pollen fertility.

In this study, *DTX35*-silenced plants had lower anthocyanin and flavonol contents and reduced pollen fertility, but the change in anthocyanin content was not statistically significant. This result shows that *DTX35* is mainly responsible for flavonol transport rather than anthocyanin transport and that flavonols are important for pollen fertility, consistent with previous studies [33]. In addition, previous studies have shown that anthocyanins give plants a purple color and play an important role in reducing ROS accumulation induced by abiotic stresses [43–45]. However, a few studies have shown that the absence of anthocyanins in anthers leads to reduced pollen fertility. *DFR* is an anthocyanin biosynthesis gene [10], and its silencing reduced anther anthocyanin content without significantly altering flavonol content; the content of H_2_O_2_ and MDA increased, and pollen fertility decreased. This result suggests that anthocyanins may also maintain pollen fertility by controlling ROS homeostasis.

In *Arabidopsis*, flavonoid transporter genes such as *DTX35* encode a variety of drug and toxin efflux family transporters. The content of glycosylated flavonols is reduced in *Arabidopsis DTX35* mutant plants; the anthers do not dehisce normally, and the pollen fertility is decreased [33]. In addition, as an essential chloride channel for turgor regulation in *Arabidopsis*, *DTX35* promotes the influx of anions into vacuoles during cell expansion, and its mutant produces short root hairs and pollen tubes [46]. Here, the content of flavonols was significantly reduced in anthers of *CaDTX35*-silenced plants; the level of active oxygen increased, pollen vigor and germination rate decreased, and the pollen tube length was significantly shorter than that of control plants. These results show that *CaDTX35* may have similar functions to *AtDTX35*. *DTX29*, *DTX30*, *DTX31*, *DTX33*, *DTX34*, and *DTX35* are from the same evolutionary lineage of MATE family proteins, and most members of this branch have important functions in regulating the elongation tip growth of plant cells [47]. For example, previous studies have shown that *DTX30*, *DTX31*, *DTX33*, and *DTX35* are involved in the regulation of root hair number and length in *Arabidopsis* [46, 48, 49]. Here, we have confirmed that *CaDTX35* plays an important role in pollen germination and pollen tube elongation. Whether other members of this evolutionary lineage have the same function during pollen germination is a worthwhile topic for future study.

In summary, our study showed that CabHLH1 regulates flavonoid synthesis by positively regulating the expression of *DTX35* and *DFR*, thereby acting as a positive regulator of pepper pollen fertility. This study provides new insights into the regulatory mechanism of flavonoid synthesis in pepper anthers and confirms the important effects of flavonoids on pepper pollen fertility.

## Materials and methods

### Plant materials and growth conditions

Wild-type (WT) and *rpf1* plants were grown in growth chambers with a 16-hour light (25°C)/8-hour dark (18°C) photoperiod cycle and 65% relative humidity. Different tissues, including leaf, root, stem, pericarp (color break), seed, placenta, and bud (pre-flowering), were gathered from maturing plants for expression analysis. Pepper anthers were collected at four developmental stages: pollen mother cell (heart leaves unfold and calyx tightly wraps corolla); tetrad (calyx slightly split and corolla slightly exposed); mononucleate microspore (the part of the corolla that extends from the calyx is about half of the length of the calyx); and mature pollen (the part of the corolla that extends out of the calyx is as long as the calyx).

### Pollen vigor analysis

The pollen from five flowers was shaken onto a glass slide, 1% iodine–potassium iodide (I_2_-KI) staining solution was added, a cover glass was placed over the sample, and the staining was observed and photographed under a microscope. Six fields of view were randomly selected in each sample for observation of pollen vigor. The *in vitro* pollen germination experiment was performed as described previously with slight modifications [15]. Culture medium was prepared with 120 mg/ml sucrose, 0.05 mg/ml H_3_BO_3_, 0.3 mg/ml Ca(NO_3_)_2_·4H_2_O, 0.2 mg/ml MgSO_4_·7H_2_O, and 0.1 mg/ml KNO_3_, and the pH was adjusted to 6.5 with 2 M KOH. Pollen from freshly opened pepper flowers was spread evenly onto the slides with a drop of culture solution, then cultured for 3 hours in the dark at 28°C; pollen germination was observed and photographed under a microscope.

### Electron microscopy, semi-thin sectioning and transmission electron microscopy

For electron microscopy, the anthers of a fully opened pepper flower were shaken with tweezers to spread the pollen evenly on a metal stage with double-sided tape. At the same time, the anthers were also fixed on the metal table. The pollen grains and anthers were sprayed with gold powder for 4–5 minutes using an Eiko IB5 ion jet (Japan), and pollen morphology was then observed using a Hitachi Model TM-1000 scanning electron microscope (Japan).

For semi-thin section observation, the anthers of WT and *rpf1* plants at different developmental stages were fixed in 1% osmic acid prepared in 0.1 M phosphate buffer (pH 7.4) for 7 hours and dehydrated in an ethanol concentration gradient. The anthers were then infiltrated with a mixed gradient solution of acetone and 812 embedding medium and embedded in spur resin. Semi-thin sectioning was performed with a Leica HistoCore Nanocut R microtome (Leica, Germany), and sections were stained with toluidine blue solution (Servicebio, G1032). An Olympus BX51 digital microscope (Japan) was used for observation of the semi-thin sections. For transmission electron microscopy (TEM) observation, ultra-thin sectioning was performed with a Leica UC7 microtome (Leica, Germany), and sections were stained with toluidine blue solution. Images were photographed with a Hitachi HT7800 (Japan) transmission electron microscope.

### *In situ* hybridization

Anthers were fixed in FAA (50% ethanol, 10% formaldehyde [37%], 5% acetic acid) overnight at 4°C, dehydrated in an ethanol series, and embedded in Paraplast High Melt paraffin (Leica) for sectioning. The *in situ* hybridization was performed as described previously [16] and transcribed *in vitro* to create probes using the Digoxigenin RNA Labeling Kit (Roche). The primers are listed in Supplementary Data Table S1.

### Determination of anthocyanin, flavonol, and flavonoid contents

Anthocyanin content was determined by a previously described method [17]. In brief, 0.2 g of anther tissue was ground in liquid nitrogen. The powdered sample was extracted with 1% acidic methanol at room temperature for 18 hours in the dark and centrifuged at 12 000 rpm for 1 minute. The supernatant (400 μl) was mixed with 600 μl acidic methanol, and the absorbance of the mixture was measured at 530 nm (A530) and 657 nm (A657). The anthocyanin content was calculated as (A530 – 0.25 × A657) g^−1^ fresh weight. A flavonoid detection kit (A142-1-1, Nanjing Jiancheng Institute of Bioengineering) was used to determine flavonoid content, and flavonol compounds were detected by high-performance liquid chromatography (HPLC) as described previously [18].

### Determination of H_2_O_2_ and malondialdehyde contents

Malondialdehyde (MDA) content and H_2_O_2_ content of anther tissues were measured using kits A003-1-2 and A064-1-1, respectively, from Nanjing Jiancheng Institute of Biological Engineering, following the manufacturer’s instructions.

### Plant treatments

DPI was applied to pepper buds as described previously with slight modifications [9]. Before blooming, a DMSO solution containing 10 μM DPI was sprayed onto the flower buds seven times at intervals of 1 day, and the same concentration of DMSO was sprayed as a control. Twenty fruits were randomly selected from each treatment, and the numbers of seeds in the fruits were counted.

### Transcriptome sequencing

Total RNA was extracted using the TRIzol method from three replicate anther samples of WT ‘8214’ and mutant *rpf1* plants just before opening, and total RNA integrity was measured on an Agilent 2100 Bioanalyzer using an RNA Nano 6000 Assay kit. Oligo(dT) magnetic beads were used to enrich mRNA, which was then fragmented using fragmentation buffer. After reverse transcription into cDNA, AMPure XP beads were used for purification and repair, and appropriate size fragments were selected for PCR enrichment to construct a cDNA library for sequencing on the Illumina HiSeq 4000 platform. After filtering out reads that contained adapters and poly-N and removing low-quality reads, we calculated the Q20, Q30, and GC content of the clean reads. Tophat2 v2.1.1 [19] was used to align the reads to the pepper reference genome [20], and edgeR v3.18.1 [21] was used to identify differentially expressed genes (DEGs). The DEGs were subjected to GO (Gene Ontogeny) enrichment analysis using the clusterProfiler v3.6.0 R package [22] and KEGG (Kyoto Encyclopedia of Genes and Genomes) pathway enrichment analysis using KOBAS 2.0 software [23].

### Gene expression analysis

Total RNA was isolated from pepper anthers according to the instructions of an RNA Isolation Kit (Huayueyang, Beijing, China). RNA was then reverse transcribed into cDNA using a first-strand cDNA Synthesis Kit (Vazyme). qRT–PCR was performed as described previously [24]. The primers are listed in Supplementary Data Table S1.

### Genetic analysis

The WT pepper cultivar ‘8214’ has normal pollen fertility and purple anthers. A mutant with lower pollen fertility, *rpf1*, was obtained from its progeny by ethyl methanesulfonate (EMS) mutagenesis, and its traits were stably inherited after multiple generations of selfing. We crossed WT ‘8214’ and *rpf1* as parents to obtain an *F*_1_ population and then performed self-crossing to obtain the *F*_2_ population. At the same time, *F*_1_ and *rpf1* were backcrossed to obtain the BC_1_ population. All the plants were planted in a greenhouse at Hunan Agricultural University. Pollen vigor was assessed for each plant in each generation, and the *χ*^2^ test was used to determine the pattern of genetic inheritance of the mutant pollen trait.

### Candidate gene mapping

A modified MutMap [25] method was used to determine the target gene candidate interval. We selected 30 WT individuals and 30 mutant individuals from the *F*_2_ segregated population and also sampled the WT ‘8214’. The modified CTAB method [26] was used to extract leaf genomic DNA, and two mixed pools were constructed and sequenced on the Illumina HiSeq 2500 platform for whole-genome resequencing. The reads were aligned to the pepper reference genome [20] using BWA software v0.7.12 [27], and SAMtools v1.5 [28] was used to identify single-nucleotide polymorphism (SNP) sites across the whole genome. The SNP filtering criteria were base quality ≥30, mapping quality ≥30, base depth in the two *F*_2_ mixed pools ≥2 and ≤ 80, and base depth in the parent ≥2 and ≤ 40. SNPs that were not typical EMS-mutagenized-type mutations (C to T or G to A) were also filtered out. ANNOVAR (v2018Apr16) [29] was used for gene annotation. High-quality polymorphic SNPs from the offspring pools were compared with the WT reference sequence to calculate the SNP index [30]. The SNP index of the two offspring pools was calculated using a 10-SNP sliding window with a step size of 5 SNPs. The statistical 99% confidence intervals of the *Δ*SNP index were calculated following a previously described method [31].

KASP genotyping analysis was used to detect the co-segregation of each SNP and identify candidate genes. Allele-specific primers were ligated to the fluorescent dyes HEX and FAM at their 5′ ends, and the genotype was detected by reading the fluorescent signal. 571 *F*_2_ generation plants, including 139 mutant plants and 432 plants with the WT phenotype, were used for KASP genotyping. The allele-specific primers are listed in Supplementary Data Table S1.

### CabHLH1 subcellular localization

The coding sequence of *CabHLH1* without the stop codon was inserted into the pSuper1300 vector to generate the recombinant expression vector pSuper1300-CabHLH1-GFP. The recombinant plasmid was transformed into *Agrobacterium* strain GV3101 by the heat shock method. pSuper1300-CabHLH1-GFP and the nuclear marker HY5-mCherry were then transiently co-transformed into *Nicotiana benthamiana* leaves. Two days later, the fluorescence was visualized and photographed with a confocal laser scanning microscope (LSM800, Zeiss, Germany).

### Virus-induced gene silencing

The online SGN VIGS tool (https://vigs.solgenomics.net/) was used to identify specific regions of *CabHLH1*, *CaDFR*, and *CaDTX35*. Based on the EcoRI and BamHI restriction sites in the pTRV2 vector, a specific primer was designed to amplify pepper anther cDNA and obtain a specific product with a length of ~300 bp (Supplementary Data Table S1). This fragment was ligated into the pTRV2 vector by a double enzyme digestion method and transferred into *Agrobacterium* GV3101. With reference to a previous method, the pTRV1 bacterial solution was mixed 1:1 with a bacterial solution containing pTRV2, pTRV2-*CabHLH1*, pTRV2-*CaDFR*, or pTRV2-*CaDTX35* to prepare for inoculation. Seedlings of the pepper variety ‘S8’ were grown until the two cotyledons had fully expanded but true leaves had not yet grown. The needle-free infiltration method was used to inoculate the pepper cotyledons after a 2-day dehydration treatment. After inoculation, the plants were cultivated in the dark at 18°C for 3 days in an artificial climate box. The growth conditions were adjusted to a day/night temperature of 22°C/18°C, a light level of 375 μmol m^−2^ s^−1^ (16 hours light/8 hours dark), and a relative humidity of 60%, and the plants were grown for ~4 weeks.

### Yeast one-hybrid and dual-luciferase reporter assays

To study the interaction between the CabHLH1 transcription factor and the flavonoid synthesis and transport-related genes *DTX35* and *DFR*, the WT *CabHLH1* and mutant *CabHLH1*-mut open reading frame (ORF) sequences were inserted into the pGADT7 vector and used as the prey proteins. The *DTX35* and *DFR* promoter sequences containing G-box elements were inserted as bait into the pAbAi vector. The bait plasmids pAbAi-*DFR* and pAbAi-*DTX35* were linearized and transformed into the Y1H Gold yeast strain, and bait strains with empty pGADT7 vectors were screened to determine the minimum inhibitory concentration of aureobasidin A (AbA). Then pGADT7-CabHLH1 and pGADT7-CabHLH1-mut were transformed into bait strains, grown on SD/−Leu medium containing the lowest inhibitory concentration of AbA, and observed after culturing at 30°C for 3 days. The ORF sequences of *CabHLH1* and *CabHLH1*-mut were also inserted into the pGreenII 62-SK-GFP vector to generate the effector plasmid. The promoters of the flavonoid synthesis-related genes *DFR* and *DTX35* were inserted into the pGreenII 0800-LUC vector to drive the expression of the LUC reporter gene and used as the reporter plasmid. These vectors were transformed into *Agrobacterium* strain GV3101. The reporter and effector plasmids were mixed at a ratio of 1:10 (v/v), and the mixed *Agrobacterium* strain GV3101 was then injected into young tobacco leaves. Two to three days after injection, the tobacco leaves were harvested for fluorescence signal observation and LUC activity detection. A Tanon 5200 imaging system was used to collect fluorescence signal images, and a GloMax 96 microplate luminescence detector (Promega) was used to detect the fluorescence values of LUC and REN (*Renilla* luciferase). There were at least six biological replicates per sample. The primer pairs used for Y1H and dual-LUC assays are listed in Supplementary Data Table S1.

## Supplementary Material

supp_data_uhac172

## Data Availability

The raw data are accessible at NCBI under the BioProject PRJNA855900.
