# Supplementary material for: The bHLH1-DTX35/DFR module regulates pollen fertility by promoting flavonoid biosynthesis in Capsicum annuum L
Source: Hortic Res. 2022 Aug 3;9:uhac172. doi: 10.1093/hr/uhac172 (PMC9552195; doi:10.1093/hr/uhac172)
Supplement: supp_data_uhac172 [file supp_data_uhac172.zip › Supplement Figures.docx]

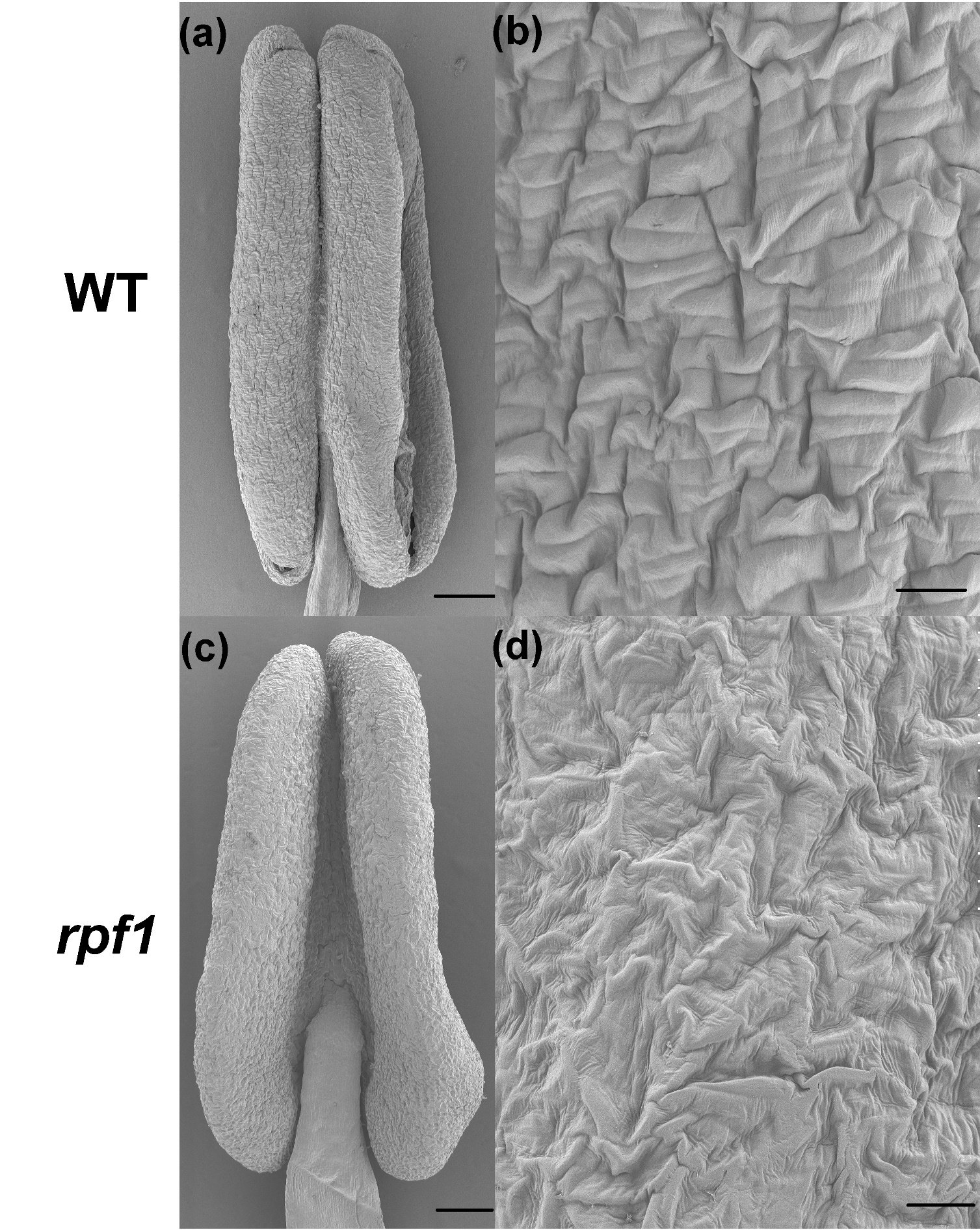


**Fig. S1 a, c** Scanning electron micrographs analysis of WT and *rpf1* anthers. **b, d** Closer views of anthers indicating pronounced changes of *rpf1* anther surface. Scale bars=300 μm in **a, c.** Scale bars=20 μm in **b, d.**

**.**


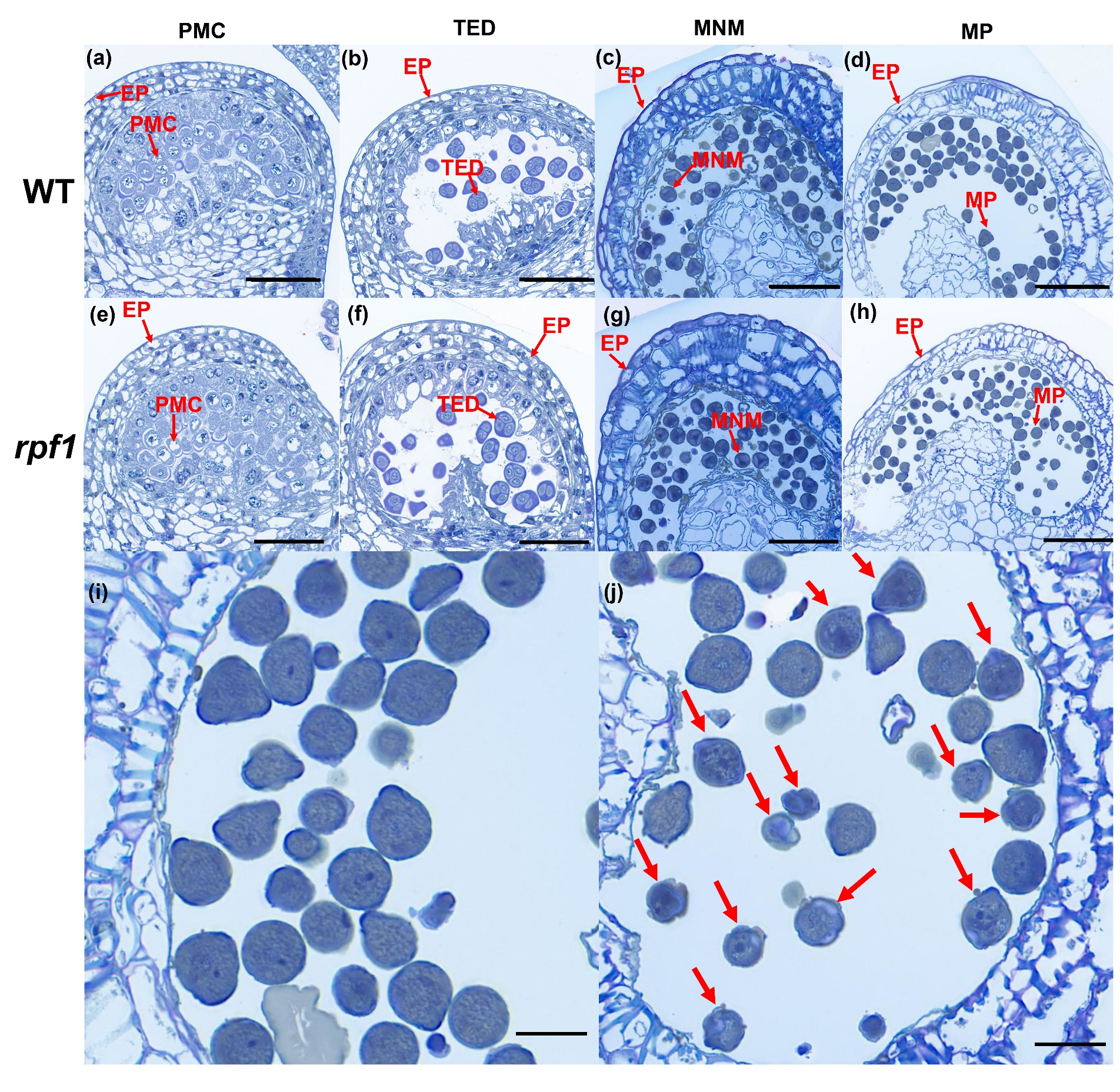


**Fig. S2 a-j** Semi-thin sections of WT and *rpf1* anthers at different developmental stages. **i** and **j** are enlarged images of **d** and **h**, respectively PMC (pollen mother cell), TED (tetrad), MNM (mononucleate microspores), and MP (mature pollen). Scale bars=50 μm in **a, b, e, f.** Scale bars=200 μm in **c, d, g, h.** Scale bars=25 μm in **i, j.**

**
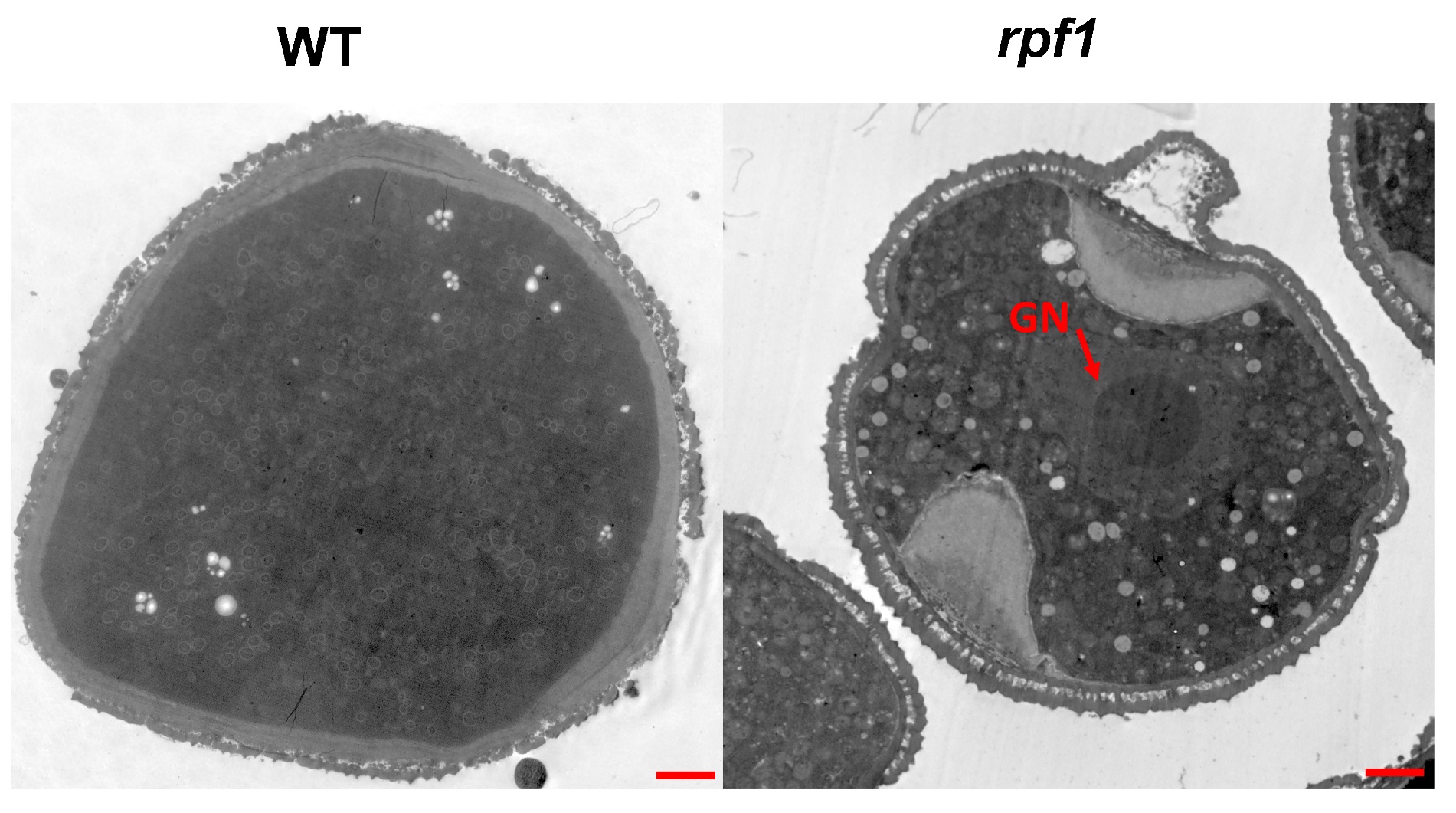
**

**Fig. S3** Transmission electron microscopy analysis of WT and *rpf1* pollen grains. Scale bars=2 μm. GN, generative nucleus


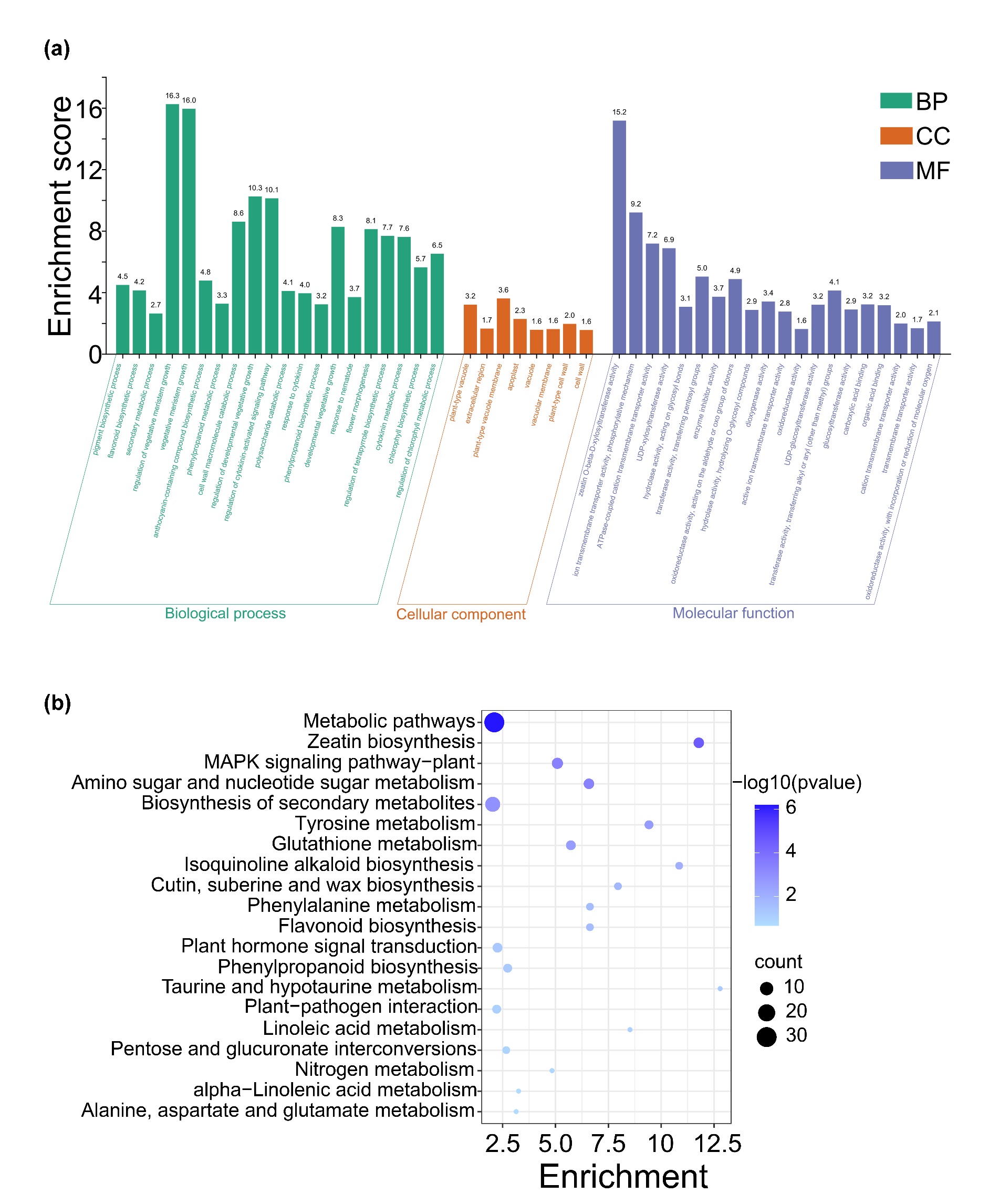


**Fig. S4 Analysis of differentially expressed genes (DEGs) in WT and *rpf1* libraries. a** GO enrichment analysis of the DEGs. **b** KEGG enrichment analysis of the DEGs.





**Fig. S5** qRT-PCR of cell wall macromolecule catabolic process and plant hormone signal transduction genes in WT and *rpf1* anthers. The values are presented as the mean ± SE (*n* = 3). “**” indicate statistically significant differences with *P* < 0.01.


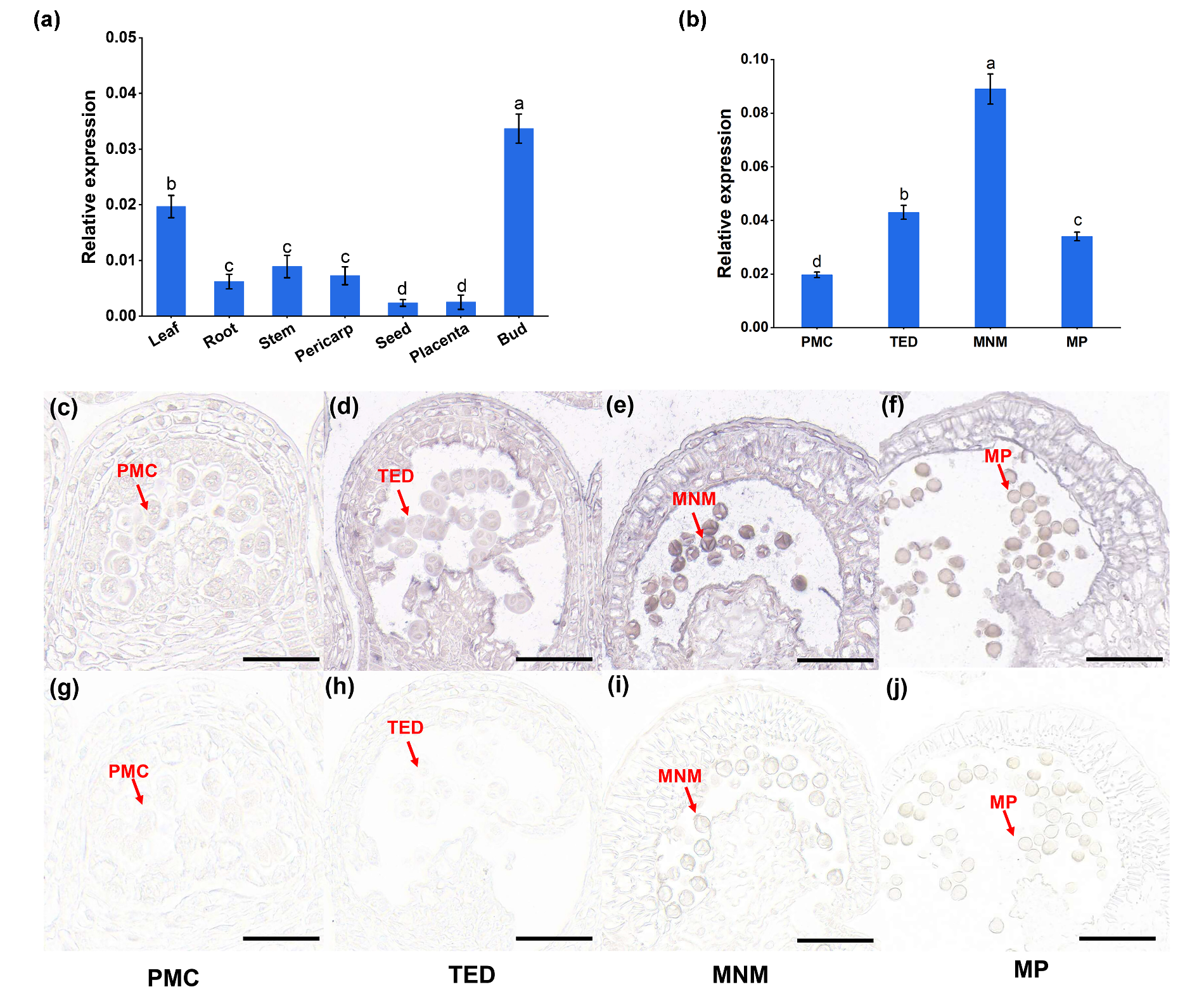


**Fig. S6 Spatial and temporal expression pattern of *CabHLH1* in pepper. a** Expression analysis of *CabHLH1* in different tissues: Leaf, Root, Stem, Pericarp, Seed, Placenta and Bud. **b** Expression analysis of *CabHLH1* in four developmental stages of stamens. PMC (pollen mother cell), TED (tetrad), MNM (mononucleate microspores), and MP (mature pollen). **c-f** Transcript levels of *CabHLH1* in pepper anthers detected by using *in situ* hybridization. **g-j** Sections of anthers at the four developmental stages, hybridized with a *CabHLH1* sense probe as a negative control. Scale bars=50 μm in **c, d, g, h.** Scale bars=200 μm in **e, f, i, j.** Lowercase letters indicate statistically significant analysis, *P* < 0.05. Data are represented as mean ± SEs (n = 3).


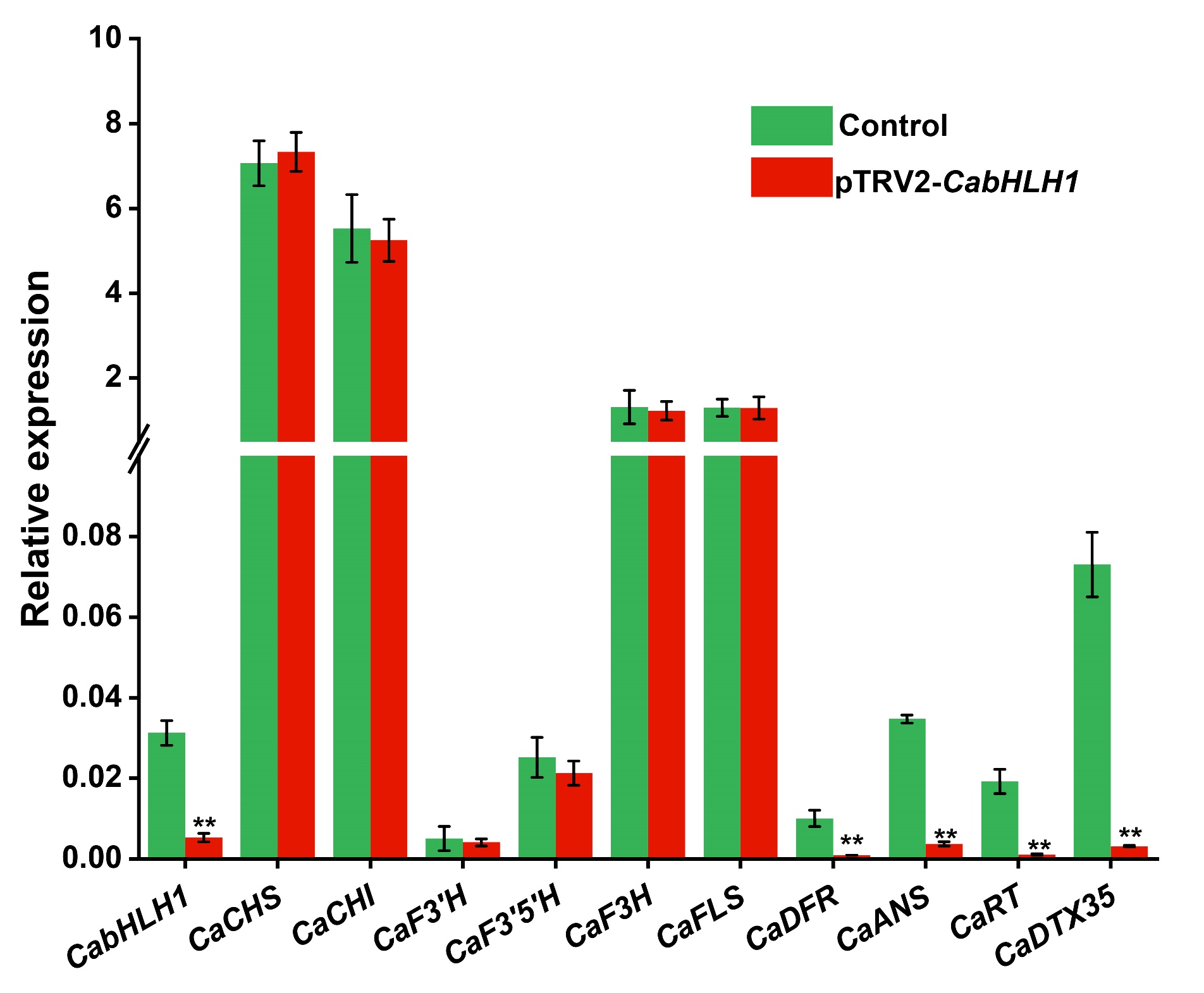


**Fig. S7 qRT-PCR of *CabHLH1* and flavonoid pathway genes in control and *CabHLH1*-silenced anthers.** The values are presented as the mean ± SE (*n* = 3). “**” indicate statistically significant differences with *P* < 0.01.


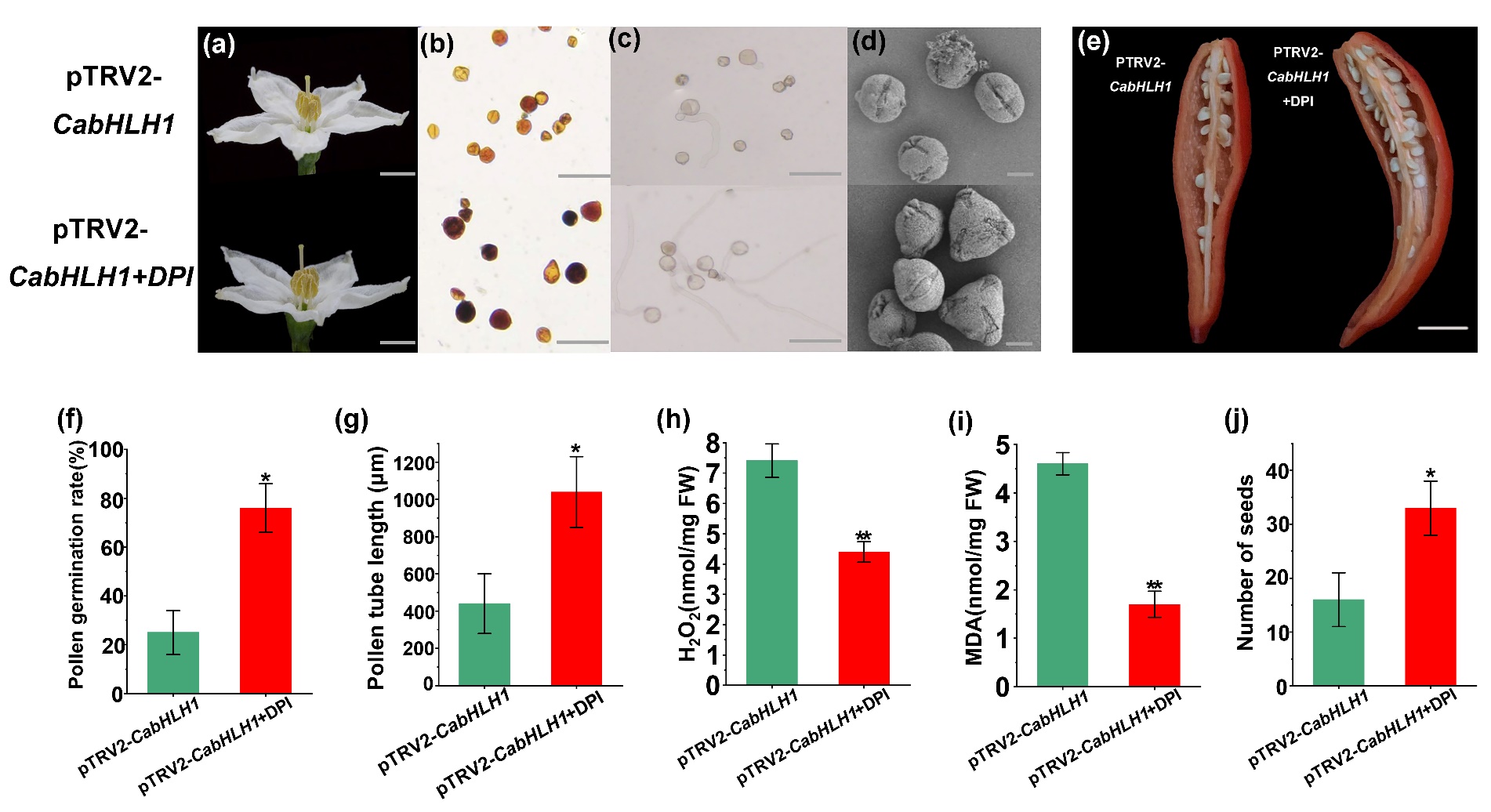


**Fig. S8 Phenotypes of** **pTRV2-*CabHLH1* and pTRV2-*CabHLH1*+DPI plants.**

**a** Flower phenotypes of pTRV2-*CabHLH1* and pTRV2-*CabHLH1*+DPI plants. Scale bars, 1 cm. **b** Pollen viability of pTRV2-*CabHLH1* and pTRV2-*CabHLH1*+DPI plants. Pollen grains in control plants were stained black by KI-I_2_ stain. Scale bar, 100 μm. **c** Pollen grains of pTRV2-*CabHLH1* and pTRV2-*CabHLH1*+DPI plants were germinated in vitro. Scale bar, 100 μm. **d** Scanning electron microscope (SEM) images of pTRV2-*CabHLH1* and pTRV2-*CabHLH1*+DPI pollen grains. Scale bar, 10 μm. **e** seeds in pTRV2-*CabHLH1* and pTRV2-*CabHLH1*+DPI fruits. Scale bar, 1 cm. **f** Pollen germination rate. **g** Pollen-tube length. **h** H_2_O_2_ content. **i** MDA content. **j** Quantification of seed set. Error bars indicate means ± SEs (n = 6) in **f** and **g**. Error bars indicate means ± SEs (n = 3) in **h** and **i**,. Error bars indicate means ± SEs (n = 20) in **j**. “*” and “**” indicate statistically significant differences with P < 0.05 and P < 0.01, respectively


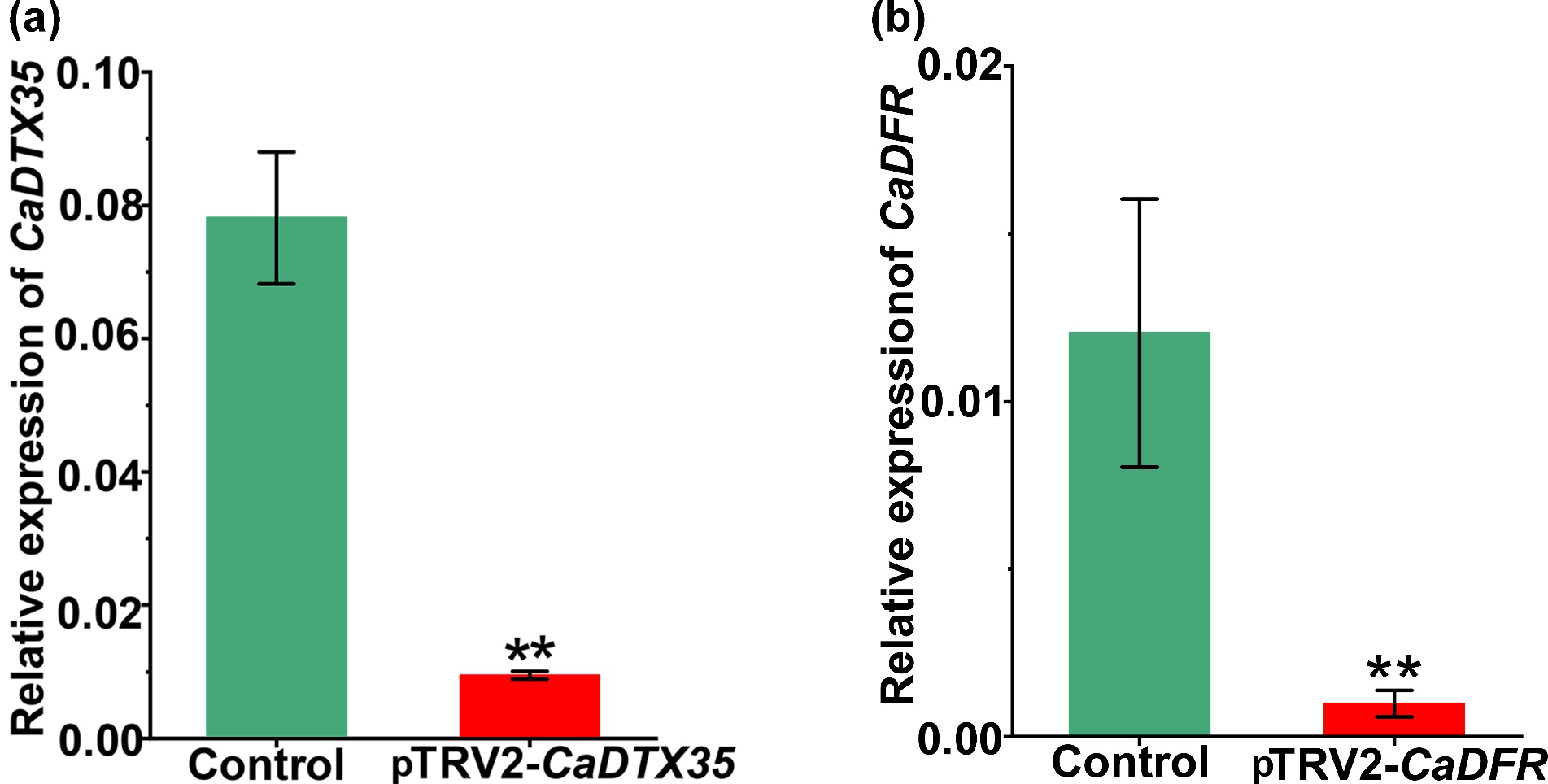


**Fig. S9 a** Relative expression of *CaDTX35* in pTRV2-*CaDTX35* anthers. **b** Relative expression of *CaDFR* in PTRV2-*CaDFR* anthers. The values are presented as the mean ± SE (*n* = 3). “**” indicate statistically significant differences with *P* < 0.01.
